# Accuracy and Impact on Patient Management of New Tools for Diagnosis of Sepsis: Experience with the T2 Magnetic Resonance Bacteria Panel

**DOI:** 10.3390/pathogens10091132

**Published:** 2021-09-02

**Authors:** Riccardo Paggi, Elio Cenci, Giuseppe Vittorio De Socio, Alessandra Belati, Daniele Marini, Alessio Gili, Barbara Camilloni, Antonella Mencacci

**Affiliations:** 1 Medical Microbiology Section, Department of Medicine, University of Perugia, Polo Unico Sant’Andrea delle Fratte, 06132 Perugia, Italy; paggi.riccardo@gmail.com (R.P.); elio.cenci@unipg.it (E.C.); alessandra.belati.93@gmail.com (A.B.); danielemarini92@gmail.com (D.M.); barbara.camilloni@unipg.it (B.C.); 2 Clinic of Infectious Diseases, Perugia General Hospital, 06129 Perugia, Italy; giuseppe.desocio@ospedale.perugia.it; 3 Public Health Section, Department of Experimental Medicine, University of Perugia, Piazza Lucio Severi 1, 06132 Perugia, Italy; alessio.gili@unipg.it

**Keywords:** sepsis diagnosis, T2 magnetic resonance, bacteria, blood culture, directed therapy

## Abstract

The rapid and accurate identification of pathogens responsible for sepsis is essential for prompt and effective antimicrobial therapy. Molecular technologies have been developed to detect the most common causative agents, with high sensitivity and short time to result (TTR). T2 Bacteria Panel (T2), based on a combination of PCR and T2 magnetic resonance, can identify directly in blood samples *Escherichia coli*, *Staphylococcus aureus*, *Klebsiella pneumoniae*, *Pseudomonas aeruginosa*, *Enterococcus faecium*, and *Acinetobacter baumannii* pathogens. This study evaluates the role of T2 in the diagnosis of sepsis and its impact on patient management, specifically in terms of TTR and the switch from empirical to directed therapy, comparing results of blood culture (BC) and T2 assay in 82 patients with sepsis. T2 significantly improved the detection of the causative agents of sepsis. For pathogens included in the panel, T2 sensitivity was 100% (95% CI 86.3–100.0), significantly higher than that of BC (54.8%, 95% CI 36.0–72.7). The TTR (median, IQR) of positive T2 (3.66 h, 3.59–4.31) was significantly shorter than that of the positive BC (37.58 h, 20.10–47.32). A significant reduction in the duration of empiric therapy and an increase in the percentage of patients with switched therapy was observed in patients with a positive T2 result. In conclusion, T2 can shorten and improve the etiological diagnosis of sepsis with a positive impact on patient management.

## 1. Introduction

Sepsis is a life-threatening organ dysfunction caused by a dysregulated host response to infection [1] and is associated with high morbidity and mortality [2,3]. A delay on the appropriate antimicrobial therapy is a strong and independent predictor of poor outcome in septic patients [4,5,6]. Broad-spectrum empiric antimicrobial therapy is recommended to minimize the risk of inadequate initial treatment and associated mortality [7] but may not be appropriate in infections due to multidrug resistant organisms (MDRO). These infections have dramatically increased in the last two decades [8,9] and are burdened by high mortality [10,11]. Infections from different anatomic foci can lead to sepsis, which is a consequence of clinical bacteremia [12]. Blood culture (BC) is considered the gold standard for sepsis etiological diagnosis [13], endowed with good sensitivity for cultivable viable pathogens [14], but suffering usually of a rather long turnaround time and substantial delay or even failure to detect microorganisms in patients already treated with antimicrobials. Moreover, microbial load can be as low as 1–10 colony forming unit/mL, a value that can result in a false negative BC [15].

Rapid and accurate microbiological diagnosis is one of the major targets to guide timely and effective antimicrobial therapy. Several technologies have been developed to rapidly identify the most common pathogens and/or antimicrobial resistance mechanisms implicated in infections [13]. Next-generation sequencing technology, based on unbiased sequence analyses of free circulating DNA, has been employed in critically ill patients with sepsis, with a complete workflow of about 30 h [16]. This technology showed higher sensitivity and specificity than BC, and could be useful for a more appropriate therapy in septic patients [17]. Polymerase-chain-reaction (PCR) methods, applied directly on whole blood, showed sensitivity higher than BC, a shorter time-to-result (TTR), and are not affected by empirical antimicrobial treatment [18,19,20]. One of the most employed RT-PCR-based systems is the LightCycler® SeptiFast (Roche Diagnostics GmbH, Mannheim, Germany), which was commercialized in 2004 and discontinued at the end of 2019 [20]. Sensitivity was sub-optimal in unselected patient populations [21,22], but significantly higher than BC in antibiotic treated patients [21,23]. Specificity reached 100% in some studies [21]. The use of this assay was associated with higher rates of antibiotic changes, with reduction of inadequate antibiotic therapy [20,24]. Nevertheless, the test was not very successful on the market, requiring a large laboratory commitment, and its cost effectiveness was not unequivocally demonstrated.

The role of other, recently released, molecular diagnostic instruments, such as the T2 Magnetic Resonance (T2MR) system, represents a current issue that deserves to be investigated. T2MR system (T2Dx®, T2 Biosystems, Lexington, MA, USA.) uses specific panels in order to identify the most prevalent and deadly bacterial and fungal pathogens, directly from whole-blood samples. It employs a fully automated process involving PCR and probe-enriched superparamagnetic nanoparticles [25]. It has been demonstrated that T2 Candida panel, compared to BC, may shorten times of *Candida* detection, with a sensitivity higher than BC, especially during antifungal therapy, so that a negative result may exclude active candidemia [26,27,28,29]. A recent study, performed in a large community hospital, found that T2 Candida panel allowed faster initiation of antifungal treatment compared to BC, but it did not improve the length of stay or mortality [30]. T2 bacterial panel (T2) is designed to enable detection of *Escherichia coli*, *Staphylococcus aureus*, *Klebsiella pneumoniae*, *Pseudomonas aeruginosa*, *Enterococcus faecium*, and *Acinetobacter baumannii* bacteria from a single whole-blood sample. Thus, the panel includes pathogens with high probability of being MDRO, representing six among the ten most common bacterial species responsible of bloodstream infections [12,31]. Few studies evaluated the impact on patient management and therapy of the implementation of T2MR technology in the microbiological diagnosis of sepsis. In a recent systematic review by Giannella et al., the use of T2MR was associated with shorter time to detection and species identification, shorter time to directed therapy, and shorter length of hospital and intensive care unit stay [32].

The aim of the present study was to evaluate the accuracy of the T2MR system for the diagnosis of bacterial sepsis and its impact on patient management, in terms of duration of empirical therapy and switch to directed therapy.

## 2. Results

### 2.1. Study Population and Antimicrobial Therapy at the Time of T2 Sample Collection

During the study period, from a total of 82 patients, 82 BC and 82 T2 samples were collected. The main characteristics of the study population are summarized in Table 1. In total, 69 out of 82 (84.1%) patients were already under antibiotic treatment, 11 patients started the antimicrobial therapy soon after blood sampling, and 2 were not given antimicrobials at all. Among treated patients, 7 received antibiotic monotherapy, and the others a combination of 2 (31 patients), 3 (25 patients), 4 (13 patients), and 5 (4 patients) molecules. Broad-spectrum antibiotics (carbapenems and piperacillin/tazobactam) were empirically administered to 48/82 (58.5%) patients (34 meropenem, 9 piperacillin/tazobactam, 4 ertapenem, 1 imipenem). Glycopeptides were administered to 17/82 patients (11 vancomycin and 6 teicoplanin) and aminoglycosides to 16/82 patients (15 amikacin and 1 gentamycin).

### 2.2. Comparison of T2 and BC Results

Concordant T2/BC results were obtained in 62/82 (75.6%) patients; 52/82 (63.4%) were concordant negative and 10/82 (12.2%) concordant positive (Table 2).

T2+/BC− discordant results were observed in 14/82 patients: 12 cases (14.6%) due to true pathogens and 2 cases to contaminated samples. Thus, the etiology of infection was identified by both methods (T2 and BC) in 10 patients, by T2 alone in 12 patiens, and by BC alone in 6 patients. The use of T2 resulted in an increased rate of pathogen detection, as the combination of the two methods allowed the etiological diagnosis in 28 cases (34.1%), and BC in 16 (19.5%) (*p* < 0.001).

Overall, 31 pathogens were identified, 25 of which belong to species included in the T2 panel and 6 to other species. The molecular test detected 25/25 pathogens included in T2 panel (4 *E. coli*, 4 *S. aureus*, 9 *K. pneumoniae*, 7 *P. aeruginosa*, and 1 *E. faecium*), a number significantly higher (*p* < 0.001) than BC, detecting 11/25 pathogens (1 *E. coli*, 1 *S. aureus*, 6 *K. pneumoniae*, 2 *P. aeruginosa*, and 1 *E. faecium*). No false negative result was observed for T2, while BC failed to identify 14 organisms. Three cases of polymicrobial sepsis were observed: two cases (one from *K. pneumoniae* and *P. aeruginosa*, and another from *S. aureus* and *P. aeruginosa* organisms) were detected only by the T2MR system. Moreover, one from *K. pneumoniae* and *E. faecium* pathogens was identified by both methods.

Six extra-panel pathogens were isolated by BC: *Serratia marcescens* (2), *Enterobacter cloacae* (1), *Bacillus clausii* (1), *Nocardia farcinica* (1), and *Staphylococcus epidermidis* (1).

Four organisms were considered contaminants according to the criteria specified in Material and Methods: 3 detected by T2 (2 *A. baumanni* and 1 *P. aeruginosa*), and 1 isolated by BC (*Staphylococcus capitis*). In addition, 2 out of 4 contaminants were detected from samples also harboring a true pathogen. In one case, a *A. baumannii* organism, detected together with *K. pneumoniae* in a patient with previous BC sets positive for *K. pneumoniae* alone, was considered contaminant for these reasons: it was not cultured from patient blood or extra-blood specimens and it was not confirmed by a second T2 assay. In the second case, a *S. capitis* organism, isolated from BC together with *S. epidermidis* in a patient with *S. epidermidis* endocarditis, was judged a contaminant.

Considering the 6 bacterial species included in the panel, T2 sensitivity was 100.0% (95% CI, 86.3–100.0), specificity 94.6% (95%CI, 84.9–98.9), NPV 100.0%, and PPV 89.3% (95% CI, 73.2–96.2). 

Including also extra-panel species, T2 sensitivity was 80.7% (95% CI, 62.5–92.6), specificity 94.6% (95%CI, 84.9–98.9), NPV 89.7 (95% CI, 80.8–94.7), and PPV 89.3% (95% CI, 73.2–96.2).

BC sensitivity was 54.8% (95% CI, 36.0–72.7), specificity 98.1% (95/% CI, 89.9–100.0), PPV 94.4% (95% CI, 70.4–99.2), and NPV 78.8% (95% CI, 71.6–84.6).

### 2.3. Clinical and Laboratory Details in Patients with Discordant T2+/BC− Results

Table 3 shows clinical, laboratory, and antimicrobial therapy data of 12 patients in which the etiological diagnosis of sepsis was based only on T2 results. Seven out of 12 patients were male, median age was 69 years (IQR 48–78), and 50% of the patients were hospitalized in a medical ward. The origin of sepsis was unknow in 3/12 cases (25%). In 6 patients, T2 pathogens had not been cultured from any sample collected during the previous 15 days. All patients were under empirical therapy at the moment of sample collection, that was switched to a directed therapy in 8/12 cases (66.7%). Median duration of empirical therapy was 20.3 h (IQR 8.6–31.3). Further, 2 out of 12 patients (16.7%) died within 30 days from sample collection.

### 2.4. Impact on Time to Results and Therapy

**Figure 1:**
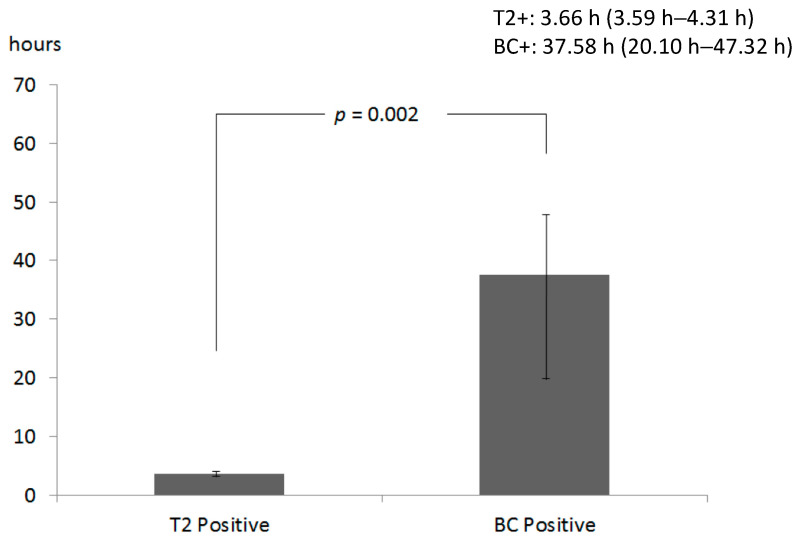
Time to Report (median, IQR) of positive T2 bacteria panel (T2) and positive blood culture (BC). Time to report was the interval between the time when a sample was collected and the time when T2 or BC results were reported.

The impact of T2 on patient management was evaluated in terms of TTR, duration of empirical therapy, and switch from empirical to directed therapy. It was found that, for a total of 82 samples, median TTR for T2 was 3.7 h (IQR 3.6–4.1) and that for BC was 116.9 h (IQR 106.8–119.6). Figure 1 shows that time to results for T2 positive samples was significantly shorter than that for positive BC (*p* = 0.002, Mann-Whitney U test).

Subsequent analyses were performed excluding 2 patients dead the same day of T2 collection, 2 patients with contaminated samples, and 6 patients with BC positive for extra-panel organisms.

**Figure 2:**
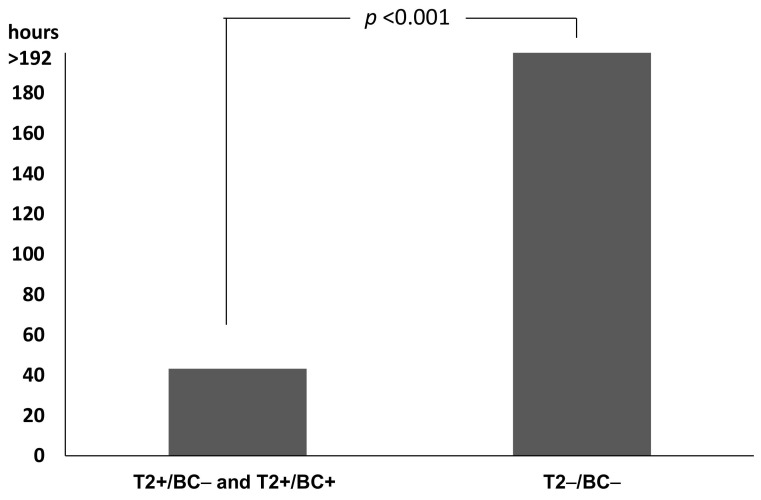
Duration of empirical therapy in 22 patients with T2 positive results (12 T2+/BC− and 10 T2+/BC+) and 50 patients with T2−/BC− results. Empirical therapy was the antimicrobial regimen started to treat patients at the time of sepsis diagnosis, directed therapy was the therapy given after knowing the etiologic agent of the infection.

Duration of empirical therapy and switch to directed therapy were evaluated in 22 patients with positive T2 results (12 T2+/BC− and 10 T2+/BC+ patients) and in 50 patients with T2−/BC− results. Maintenance of empirical therapy was arbitrarily considered as duration of empirical therapy >192 h (8 days). Duration of empirical therapy in patients with positive T2 results was significantly shorter than in patients with T2−/BC− results (*p* < 0.001, Mann-Whitney U test). In T2 positive patients (T2+/BC− and T2+/BC+) median time was 43.26 h (IQR 23.91–192.00) and for T2−/BC− patients 192 h (IQR 192.00–192.00) (Figure 2).

**Figure 3:**
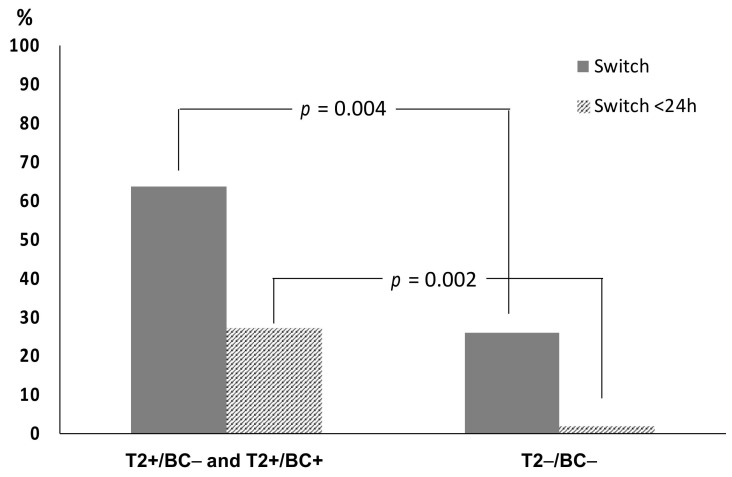
Switch from empirical to directed therapy in 22 patients with T2 positive results (12 T2+/BC− and 10 T2+/BC+) and 50 with T2−/BC− results (grey columns). Striped columns represent cases in which therapy was switched <24 h from T2 sample collection. Statistical differences were determined according to Pearson’s Chi-square test.

A significant difference in the percentage of patients whose empirical therapy was changed to directed therapy was found between groups with T2 positive results (T2+/BC− and T2+/BC+) and the T2−/BC− group. In particular, switch from empirical to directed therapy was observed in 14/22 (63.6%) T2 positive patients and in 13/50 (26.0%) T2−/BC− patients. (Figure 3). Switch within 24 h from sample collection was observed in 6/22 (27.3%) T2 positive and in 1/50 (2.0%) T2−/BC− patients (Figure 3). The switch was observed in 2/6 patients (33.3%) positive only by BC.

## 3. Discussion

The main finding of this study was that the addition of T2MR assay to BC for the diagnosis of sepsis resulted in an increased rate of pathogen detection, with 12/28 cases due to 14 pathogens diagnosed only by the molecular test. A similar result was found by Voigt et al., with T2 detecting 25% more positive samples than BC [33]. In comparison to patients with T2−/BC− results, a significant reduction of duration of empirical therapy and an increase of switch to directed therapy was observed in patients with a positive T2 result, especially in those with T2+/BC− results (66.7% switch). More than two-thirds of cases with identified etiology (22/28, 78.6%) were caused by bacterial species included in T2 panel, a finding in line with other studies [33]. A high sensitivity for this system in detection of pathogens included in the panel has been reported [34,35,36,37]. We found that T2 sensitivity was 100%, and that of BC 54.8%, a difference possibly due to the fact that the majority of patients were already on antibiotic treatment before sampling. Indeed, culture-independent molecular methods frequently yield additional positive results in culture-negative blood samples [38], improving patient outcome when used to guide antibiotic therapy [39]. Interestingly, an in-depth analysis of T2 positive results in patients with concurrent negative BC found that bacteria detected by this system were in the majority of cases true pathogens, responsible for bloodstream infections associated with the recent use of effective antimicrobial therapy [40].

Two false positive results due to *A. baumannii* species were found. These findings, together with the fact that T2 is not FDA-approved for *A. baumannii* detection, implies that every result positive for this species must be confirmed in a second sample, collected soon after the result has been obtained. In this study specificity of T2 was lower than that of BC, that was 100%, considering as true positive a BC sample in which the causative agent of endocarditis (*S. epidermidis*) was isolated together with a *S. capitis* contaminant organism. It is known that the contamination rate for blood culture vary widely between institutions from 0.6% to over 6% [41]. It is conceivable that the low BC contamination rate observed in this study could have been related to the fact that nurses underwent specific training in specimen collection prior to the implementation of T2MR technology in routine diagnostics. On the other hand, six cases of sepsis by pathogens not included in T2 panel, including one case of *S. epidermidis* endocarditis, were missed by T2. Indeed, the inability of the system to detect some organisms responsible for serious infections [42,43,44] represents a limitation of the system and highlights that molecular methods must always be used in addition to BC, that retains a crucial diagnostic role. 

The median time to report for T2 positive results (3.66 h) was significantly shorter than that observed for positive BC (37.58 h), which was an expected finding given that the test is not dependent on the time of bacterial growth. Moreover, the short TTR is due to the intrinsic characteristics of the T2MR assay, with a very limited handling time and low laboratory commitment. Among patients with positive T2 (T2+/BC− and T2+/BC+), 27.3% received directed therapy the same day of sample collection, while the percentage was significantly lower in patients with T2−/BC− results (2.0%). This finding suggests that the positivity of T2 test could greatly influence clinical decisions and this technology could be implemented in hospital antibiotic stewardship programs to reduce the use of broad-spectrum antimicrobials [45]. The results are in line with previous studies, highlighting the reduced time to species identification and the impact of positive T2 results on the switch from empirical to target therapy [32]. 

On the other hand, it seems that a negative T2 assay was not considered sufficient to switch empirical ongoing therapy, perhaps because of the limitation of the panel, that can detect only six bacterial species. Indeed, this assay is not intended to replace culture methods, that remain the unique method to isolate all culturable pathogens and to test their antibiotic susceptibility. Preliminary results suggest that a recently released T2 panel detecting the most important bacterial resistance genes directly in blood samples, should greatly enhance the potential of this technology, when included in the diagnostic and antimicrobial stewardship programs for septic patients [46]. 

There are some limitations in this study. A selection bias could be due to the fact that the T2 test was requested based on clinical judgment and not according to unequivocal inclusion and exclusion criteria. The observational study design and the limited number of patients included do not support definitive conclusions on T2MR technology’s clinical impact. Further prospective, multicenter investigations are needed to define the exact role of this technology in the diagnosis and management of sepsis. Nevertheless, the aim of the study was not to evaluate the impact of the T2MR assay in specific settings, but in a real-life clinical practice in a general hospital. Finally, given the absence of prespecified inclusion and exclusion criteria, data on mortality were not addressed. 

In conclusion, T2 in combination with BC significantly shortens and improves the etiologic diagnosis of sepsis, reducing the duration of empirical therapy and increasing switch to directed therapy in patients with positive T2 results. Detection of *A. baumannii* organisms needs to be confirmed by repeating the test on an additional whole-blood sample.

## 4. Materials and Methods

### 4.1. Study Design

This was a retrospective observational study, carried out from 1 May 2019 to 31 January 2020, including 82 consecutive patients with sepsis, defined according to the Third International Consensus Definitions for Sepsis and Septic Shock [1]. All patients, irrespective of age and sex, for whom concurrent T2 and BC samples were requested for diagnosis were included in the study. Exclusion criteria were as follows: lack of one of the two tests, samples collected at different times, lack of clinical or laboratory data to be recorded. Clinical charts were reviewed and the following data were recorded: age, gender, concomitant diseases, pathogens detected by T2 and/or BC, pathogens detected from a previous BC or from extra-blood samples within the same infectious episode (<15 days before T2 collection), clinical and laboratory findings at the time of sample collection, antibiotic therapy, duration of empirical therapy, time to switch to directed therapy, and 30-days crude mortality. 

### 4.2. Blood Culture and Standard Protocol 

The Microbiology laboratory provides diagnostic services to the 800-bed General Hospital of Perugia, Italy, serving a population of around 200,000 people. It operates from 08:00 a.m. to 08:00 p.m., Monday to Friday, and from 08:00 a.m. to 02:00 p.m. on Saturday and Sunday/holidays. Blood cultures were received inoculated into two BACTEC Plus Aerobic/F and two BACTEC Lytic/10 Anaerobic/F bottles, and incubated immediately in the BD BACTEC FX instrument (Becton Dickinson, Sparks, MD, USA). All bottles flagged positive were processed as previously described [47]. A short-term incubation method was used for positive BC processed during laboratory hours 8:00 a.m.–11:00 a.m., Monday to Friday, in that identification (ID) and antimicrobial susceptibility testing (AST) were set up after 8 h of incubation and final results were reported the following day [47]. Outside laboratory hours, ID and AST were set up after 18-h incubation, and final results were reported the following day.

### 4.3. T2 Sampling and Assay

In patients with sepsis, T2 test was requested based on the clinician’s judgement, according to the severity of clinical picture and setting. For each patient, a whole-blood sample for the T2 bacterial panel assay was collected into 4 mL K2EDTA Vacutainer blood collection tubes, from peripheral sites, with the exception of five samples that were taken from CVC, in five pediatric patients. Samples, delivered to the Clinical Microbiology laboratory during the operating time, were processed immediately according to the manufacturer’s instructions. 

### 4.4. Analysis of Clinical Impact

All patients’ data were collected and analyzed in order to evaluate the role of T2MR technology in diagnosis and management of infection. Type and time of empiric therapy, and time to switch to directed therapy were carefully analyzed in conference by investigators (infectious disease specialists and clinical microbiologists). Times were calculated using the microbiology software package Epicenter (Becton Dickinson, Franklin Lakes, NJ, USA) and the TD-Synergy Laboratory Information System (Siemens, Italy). Time to report was the interval between the time when a sample were collected and the time when T2 or BC results were reported. To evaluate the diagnostic accuracy of BC or T2 in pathogen detection, results were interpreted according to the criteria detailed below. Microorganisms detected by T2 were considered true pathogens if the result (genus and species) coincided with the result of the BC. If T2 and BC were positive for different microorganisms, or if a microorganism was detected by only one of the two tests, culture results from other samples, taken from the suspected infection site, were evaluated. If culture was positive for the same organism, it was considered a pathogen. If a microorganism was detected in only one test (T2 or BC) without culture support from other samples of the same patient, clinical chart was revised and the microorganism was considered a true pathogen if that bacterial species is generally accepted as a common etiologic agent of the patient’s diagnosed infection. In the absence of the above criteria, a microorganism detected only by T2 was considered as a contaminant if the bacterial species was not detected in a second sample, collected soon after the T2 result. Empirical therapy was defined as the antimicrobial regimen started to treat patients at the time of sepsis diagnosis, without knowing the specific etiologic agent, while directed therapy was the therapy given after knowing the etiologic agent, either maintaining or modifying empirical therapy, according to antimicrobial guidelines specific for each pathogen.

### 4.5. Statistical Analysis

Standard descriptive statistics were used to summarize data, such median, inter-quantile range (IQR), and percentage. Pearson’s Chi-square test was used for categorical variables. The nonparametric Mann-Whitney U test, adjusted with Holm correction, was used for pairwise comparison of different patient groups. Test sensitivity, specificity, Positive Predicted Value (PPV), and Negative Predicted Value (NPV) were calculated to compare T2 with BC. T2 sensitivity and specificity were calculated based on the organisms included in the T2 panel and on extra-panel bacteria. T2 and BC samples in which a contaminant organism was detected together with a true pathogen were considered as positive T2 or BC, respectively. A *p* value < 0.05 was considered for statistical significance. SPSS statistical package release 24.0 (SPSS Inc., Chicago, IL, USA) was used for all statistical analyses.

## Figures and Tables

**Table 1:** Characteristics of the study population.

Variable	Value
**Patients**	
Years, median (IQR)	62.5 (42.0–71.0)
Men	57 (69.5%)
**Hospital ward**	
Medicine	45 (54.9%)
Surgical	3 (3.7%)
Intensive Care	34 (41.5%)
**Laboratory parameters**	
Leucocytes, cells × 10^3^/mL, Median (IQR)	12.93 (3.90–18.39)
Leukocytosis, >12 × 10^3^/mL	35 (42.7%)
Leucopenia, <4 × 10^3^/mL	21 (25.6%)
Neutrophils percentage, Median (IQR)	73.8 (66.6–87.0)
CRP, mg/dL, Median (IQR)	12.1 (6.3–19.8)
PCT, ng/mL, Median (IQR)	1.18 (0.40–7.36)
Lactate, mM/L, Median (IQR)	1.50 (1.00–2.73)
**Clinical data**	
Body Temperature, °C, Median (IQR)	37.5 (36.5–38.3)
Temperature > 38 °C	23 (28.0%)
Temperature < 36 °C	3 (3.6%)
Heart Rate, beats/min, Median (IQR)	98.0 (80.0–115.0)
Mean Arterial Pressure, mmHg, Median (IQR)	85.0 (73.0–93.0)
Mean Respiratory Rate, acts/min, Median (IQR)	20.0 (16.0–24.0)
Glasgow Coma Scale Median (IQR)	14.0 (11.0–15.0)
Septic Shock	24 (29.3%)
**Sequential Organ Failure Assessment Score, Median (IQR)**	5 (3–9)
**30-Days Mortality**	25 (30.5%)
**Concomitant diseases**	
Hypertension 18	33 (40%)
History of Cardiovascular Disease	19 (23%)
Chronic renal failure	14 (15%)
Solid Malignancy	15 (18%)
Diabetes	18 (22%)
Chronic Lung Disease	11 (13%)
Transplant	14 (17%)
Chronic Liver Disease	8 (10/%)
Hematological Disease	27 (33%)

Values are number (%), unless otherwise specified. Data refer to the time of T2 and BC samples collection with the exception of mortality. CRP, C-reactive protein; IQR, interquartile range; PCT, procalcitonin.

**Table 2:** Comparison between T2 Bacteria Panel (T2) and Blood Culture (BC) results.

	BC Positive	BC Negative	Total
**T2 positive**	10 ^1^	14 ^2^	24
**T2 negative**	6	52	58
**Total**	16	66	82

^1^ This group includes 9 monomicrobial and 1 polymicrobial infections (11 pathogens). ^2^ This group includes 10 monomicrobial and 2 polymicrobial infections, and 2 contaminated samples (14 pathogens and 2 contaminant organisms).

**Table 3:** Clinical and laboratory details in patients with discordant T2BP+/BC− results.

Age, Gender	Hospital Ward ^1^	Sofa Score	WBC × 10^3^ (% Neutrophils)	Diagnosis on Admission	Suspected Infectious Focus	T2BP Result	Same Pathogen Cultured <15 Days from T2 Collection	Empirical Therapy ^2^	Switch to Directed Therapy	Hours of Empirical Therapy
54, M	ICU	2	10.910 (82.1)	Endocarditis	Cardiac valve	*S. aureus*	No	OXA, DAP	No	N.A. ^3^
67, M	BMT	7	0.28 (7.5)	Sepsis	Unknown	*E. coli*	No	MEM	MEM, AN	26.0
71, M	BMT	9	1.28 (69.5)	Sepsis	Urinary tract	*K. pneumoniae + P. aeruginosa*	Yes (urine)	CAZ/AVI, TGC	MEM, CAZ/AVI	6.3
75, F	ICU	7	69.20 (73.3)	Sepsis	Knee Prosthetic Device	*S. aureus*	Yes (synovial fluid, blood, urine, rectal swab)	OXA, RIF	DAP	61.7
86, F ^4^	MED	9	10.42 (78.2)	Septic Shock	Unknown	*P. aeruginosa*	No	AMP/SUL, TED	C/T, AN	36.6
27, F	MED	1	24.50 (87.2)	Sepsis	Unknown	*E. coli*	No	ERT, CRO	No	N.A.
81, M	MED	15	21.37 (86.6)	Tracheo-esophageal fistula	Pneumonia	*P. aeruginosa*	Yes (respiratory tract, Rectal swab)	CAZ/AVI, TGC	CAZ/AVI, AN	8.7
75, F	ICU	1	7.04 (58.6)	Wound infection	Limb Abscess	*P. aeruginosa + S. aureus*	Yes (rectal swab)	RIF, DAP, OXA, FEP	No	N.A.
60, M ^4^	MED	5	27.61 (94.5)	Cellulitis	Wound	*K. pneumoniae*	No	CC, CRO, TEC	MER, TEC	16.6
16, M	BMT	10	0.02 (-)	Fever in BMT patient	Oral Mucositis	*P. aeruginosa*	Yes (Blood)	MEM, AN. TGC, C/T	No	N.A.
92, F	MED	5	7.53 (66.3)	Sepsis	Urinary tract	*K. pneumoniae*	Yes (urine, blood, rectal swab)	DAP, TZP, CAZ/AVI	CAZ/AVI, TZP	24.0
42, M	MED	6	36.39 (76.6)	Septic shock	Urinary tract	*E. coli*	No	DAP, FF, CAZ/AVI, TZP	MEM	8.5

^1^ BMT, bone marrow transplantation unit; MED, medical ward; ICU, intensive care unit. ^2^ AMP/SUL, ampicillin/sulbactam; AN, amikacin; C/T, ceftolozane/tazobactam; CAZ/AVI, ceftazidime/avibactam; CC, clindamycin; CRO, ceftriaxone; DAP, daptomycin; ERT, ertapenem; FEP, cefepime; FF, fosfomycin; MEM, meropenem; OXA, oxacillim; RIF, rifampicin; TEC, teicoplanin; TED, telizolid; TGC, tigecycline, TZP, piperacillin tazobactam. ^3^ N.A., not applicable. ^4^ This patient died within 30 days from sample collection.

## Data Availability

The data presented in this study are available on request from the corresponding author. A part of the results reported in the present study has been presented at 31st ECCMID 2021.

## References

[1] Singer M., Deutschman C.S., Seymour C.W., Shankar-Hari M., Annane D., Bauer M., Bellomo R., Bernard G.R., Chiche J.D., Coopersmith C.M. (2016). The third international consensus definitions for sepsis and septic shock (sepsis-3). JAMA.

[2] Cecconi M., Evans L., Levy M., Rhodes A. (2018). Sepsis and septic shock. Lancet.

[3] Reinhart K., Daniels R., Kissoon N., Machado F.R., Schachter R.D., Finfer S. (2017). Recognizing sepsis as a global health priority—A WHO resolution. N. Engl. J. Med..

[4] Kumar A., Roberts D., Wood K.E., Light B., Parrillo J.E., Sharma S., Suppes R., Feinstein D., Zanotti S., Taiberg L. (2006). Duration of hypotension before initiation of effective antimicrobial therapy is the critical determinant of survival in human septic shock. Crit. Care Med..

[5] Paul M., Kariv G., Goldberg E., Raskin M., Shaked H., Hazzan R., Samra Z., Paghis D., Bishara J., Leibovici L. (2010). Importance of appropriate empirical antibiotic therapy for methicillin resistant Staphylococcus aureus bacteraemia. J. Antimicrob. Chemother..

[6] Paul M., Shani V., Muchtar E., Kariv G., Robenshtok E., Leibovici L. (2010). Systematic review and meta-analysis of the efficacy of appropriate empiric antibiotic therapy for sepsis. Antimicrob. Agents Chemother..

[7] Rhodes A., Evan L.E., Alhazzani W., Levy M.M., Antonelli M., Ferrer R., Kumar A., Sevransky J.E., Sprung C.L., Nunnally M.E. (2017). Surviving sepsis campaign: International guidelines for management of sepsis and septic shock: 2016. Intensive Care Med..

[8] Centers for Disease Control and Prevention (CDC) (2013). Antibiotic Resistance Threats in the United States. http://www.cdc.gov/drugresistance/threatreport-2013/pdf/ar-threats-2013-508.

[9] Burnham J.P., Olsen M.A., Kollef M.H. (2019). Re-estimating annual deaths due to multidrug-resistant organism infections. Infect. Control Hosp. Epidemiol..

[10] Pogue J.M., Kaye K.S., Cohen D.A., Marchaim D. (2015). Appropriate antimicrobial therapy in the era of multidrug-resistant human pathogens. Clin. Microbiol. Infect..

[11] Santoro A., Franceschini E., Meschiari M., Menozzi M., Zona S., Venturelli C., Digaetano M., Rogati C., Guaraldi G., Paul M. (2020). Epidemiology and Risk Factors Associated With Mortality in Consecutive Patients With Bacterial Bloodstream Infection: Impact of MDR and XDR Bacteria. Open Forum Infect. Dis..

[12] Holmes C.L., Anderson M.T., Mobley H.L.T., Bachman M.A. (2021). Pathogenesis of Gram-negative bacteremia. Clin. Microbiol. Rev..

[13] Peker N., Couto N., Sinha B., Rossen J.W. (2018). Diagnosis of bloodstream infections from positive blood cultures and directly from blood samples: Recent developments in molecular approaches. Clin. Microbiol. Infect..

[14] Dubourg G., Raoult D. (2016). Emerging methodologies for pathogen identification in positive blood culture testing. Expert Rev. Mol. Diagn..

[15] Werner A.S., Cobbs C.G., Kaye D., Hook E.W. (1967). Studies on the bacteremia of bacterial endocarditis. JAMA.

[16] Grumaz S., Stevens P., Grumaz C., Decker S.O., Weigand M.A., Hofer S., Brenner T., von Haeseler A., Sohn K. (2016). Next-generation sequencing diagnostics of bacteremia in septic patients. Genome Med..

[17] Grumaz S., Grumaz C., Vainshtein Y., Stevens P., Glanz K., Decker S.O., Hofer S., Weigand M.A., Brenner T., Sohn K. (2019). Enhanced Performance of Next-Generation Sequencing Diagnostics Compared with Standard of Care Microbiological Diagnostics in Patients Suffering From Septic Shock. Crit. Care Med..

[18] Opota O., Jaton K., Greub G. (2015). Microbial diagnosis of bloodstream infection: Towards molecular diagnosis directly from blood. Clin. Microbiol. Infect..

[19] Bacconi A., Richmond G.S., Baroldi M.A., Laffler T.G., Blyn L.B., Carolan H.E., Frinder M.R., Toleno D.M., Metzgar D., Gutierrez J.R. (2014). Improved sensitivity for molecular detection of bacteria and Candida in blood. J. Clin. Microbiol..

[20] D’Onofrio V., Salimans L., Bedenić B., Cartuyvels R., Barišić I., Gyssens I.C. (2020). The Clinical Impact of Rapid Molecular Microbiological Diagnostics for Pathogen and Resistance Gene Identification in Patients with Sepsis: A Systematic Review. Open Forum Infect. Dis..

[21] Pasqualini L., Mencacci A., Leli C., Montagna P., Cardaccia A., Cenci E., Montecarlo I., Pirro M., di Filippo F., Cistaro E. (2012). Diagnostic Performance of a Multiple Real-Time PCR Assay in Patients with Suspected Sepsis Hospitalized in an Internal Medicine Ward. J. Clin. Microbiol..

[22] Warhurst G., Dunn G., Chadwick P., Blackwood B., McAuley D., Perkins G.D., McMullan R., Gates S., Bentley A., Young D. (2015). Rapid detection of health-care-associated bloodstream infection in critical care using Multipathogen real-time polymerase chain reaction technology: A diagnostic accuracy study and systematic review. Health Technol. Assess..

[23] Casalta J.P., Gouriet F., Roux V., Thuny F., Habib G., Raoult D. (2009). Evaluation of the LightCycler® SeptiFast test in the rapid etiologic diagnostic of infectious endocarditis. Eur. J. Clin. Microbiol. Infect. Dis..

[24] Mongelli G., Romeo M.A., Denaro C., Gennaro M., Fraggetta F., Stefani S. (2015). Added value of multi-pathogen probe-based real-time PCR SeptiFast in the rapid diagnosis of bloodstream infections in patients with bacteraemia. J. Med. Microbiol..

[25] Pfaller M.A., Wolk D.M., Lowery T.J. (2015). T2MR and T2Candida: Novel technology for the rapid diagnosis of candidemia and invasive candidiasis. Future Microbiol..

[26] Clancy C.J., Pappas P.G., Vazquez J., Judson M.A., Kontoyiannis D.P., Thompson G.R., Garey K.W., Reboli A., Greenberg R.N., Apewokin S. (2018). Detecting Infections Rapidly and Easily for Candidemia Trial, Part 2 (DIRECT2): A Prospective, Multicenter Study of the T2Candida Panel. Clin. Infect. Dis..

[27] Sanguinetti M., Posteraro B., Beigelman-Aubry C., Lamoth F., Dunet V., Slavin M., Richardson M.D. (2019). Diagnosis and treatment of invasive fungal infections: Looking ahead. J. Antimicrob. Chemother..

[28] Muñoz P., Vena A., Machado M. (2018). T2Candida MR as a predictor of outcome in patients with suspected invasive candidiasis starting empirical antifungal treatment: A prospective pilot study. J. Antimicrob. Chemother..

[29] Mylonakis E., Clancy C.J., Ostrosky-Zeichner L., Garey K.W., Alangaden G.J., Vazquez J.A., Groeger J.S., Judson M.A., Vinagre Y.M., Heard S.O. (2015). T2 magnetic resonance assay for the rapid diagnosis of candidemia in whole blood: A clinical trial. Clin. Infect. Dis..

[30] Steuber T.D., Butler L., Sawyer A., Chappell R., Edwards J. (2021). Comparison of blood cultures versus T2 Candida Panel in management of candidemia at a large community hospital. Eur. J. Clin. Microbiol. Infect. Dis..

[31] Diekema D.J., Hsueh P.R., Mendes R.E., Pfaller M.A., Rolston K.V., Sader H.S., Jones R.N. (2019). The microbiology of bloodstream infection: 20-year trends from the SENTRY Antimicrobial Surveillance Program. Antimicrob. Agents Chemother..

[32] Giannella M., Pankey G.A., Pascale P., Miller V.M., Miller L.E., Seitz T. (2021). Antimicrobial and Resource Utilization with T2 Magnetic Resonance for Rapid Diagnosis of Bloodstream Infections: Systematic Review with Meta-analysis of Controlled Studies. Expert Rev. Med. Devices.

[33] Voigt C., Silbert S., Widen R.H., Marturano J.E., Lowery T.J., Ashcraft D., Pankey G. (2020). The T2Bacteria Assay Is a Sensitive and Rapid Detector of Bacteremia That Can Be Initiated in the Emergency Department and Has Potential to Favorably Influence Subsequent Therapy. J. Emerg. Med..

[34] De Angelis G., Posteraro B., De Carolis E., Menchinelli G., Franceschi F., Tumbarello M., De Pascale G., Spanu T., Sanguinetti M. (2018). T2Bacteria magnetic resonance assay for the rapid detection of ESKAPEc pathogens directly in whole blood. J. Antimicrob. Chemother..

[35] Maki D.G. (2019). The T2Bacteria Panel had 90% sensitivity for detecting targeted organisms, 43% for any bloodstream infection organism. Ann. Intern. Med..

[36] Nguyen M.H., Clancy C.J., Pasculle A.W., Pappas P.G., Alangaden G., Pankey G.A., Schmitt B.H., Rasool A., Weinstein M.P., Widen R. (2019). Performance of the T2Bacteria Panel for Diagnosing Bloodstream Infections: A Diagnostic Accuracy Study. Ann. Intern. Med..

[37] Drevinek P., Hurych J., Antuskova M., Tkadlec J., Berousek J., Prikrylova Z., Bures J., Vajter J., Soucek M., Masopust J. (2021). Direct detection of ESKAPEc pathogens from whole blood using the T2Bacteria Panel allows early antimicrobial stewardship intervention in patients with sepsis. Microbiologyopen.

[38] Farrell J.J., Hujer A.M., Sampath R., Bonomo R.A. (2015). Salvage microbiology: Opportunities and challenges in the detection of bacterial pathogens following initiation of antimicrobial treatment. Expert Rev. Mol. Diagn..

[39] Lodes U., Bohmeier B., Lippert H., König B., Meyer F. (2012). PCR-based rapid sepsis diagnosis effectively guides clinical treatment in patients with new onset of SIRS. Langenbeck’s Arch. Surg..

[40] Kalligeros M., Zacharioudakis I.M., Tansarli G.S., Tori K., Shehadeh F., Mylonakis E. (2020). In-depth analysis of T2Bacteria positive results in patients with concurrent negative blood culture: A case series. BMC Infect. Dis..

[41] Hall K.K., Lyman J.A. (2006). Updated review of blood culture contamination. Clin. Microbiol. Rev..

[42] Holland T.L., Baddour L.M., Bayer A.S., Hoen B., Miro J.M., Fowler V.G. (2011). Infective endocarditis. Nat. Rev. Dis. Primers.

[43] Kavanagh N., Ryan E.J., Widaa A., Sexton G., Fennell J., O’Rourke S., Cahill K.C., Kearney C.J., O’Brien F.J., Kerrigan S.W. (2018). Staphylococcal osteomyelitis: Disease progression, treatment challenges, and future directions. Clin. Microbiol. Rev..

[44] Reers Y., Idelevich E.A., Patkau H., Sauerland M.C., Tafelski S., Nachtigall I., Berdel W.E., Peters G., Silling G., Becker K. (2016). Multiplex PCR assay underreports true bloodstream infections with coagulase-negative staphylococci in hematological patients with febrile neutropenia. Diagn. Microbiol. Infect. Dis..

[45] Bogan C., Marchaim D. (2013). The role of antimicrobial stewardship in curbing carbapenem resistance. Future Microbiol..

[46] Walsh T.J., Mencacci A., Paggi R., Douka E., Vrettou C. Prospective study of T2 resistance system for detection of resistance genes in bacterial bloodsteam infections. Proceedings of the ePoster 31th ECCMID 2021.

[47] De Socio G., Di Donato F., Paggi R., Gabrielli C., Belati A., Rizza G., Savoia M., Repetto A., Cenci E., Mencacci A. (2018). Laboratory automation reduces time to report of positive blood cultures and improves management of patients with bloodstream infection. Eur. J. Clin. Microbiol. Infect. Dis..

